# Consultant doctors’ hand hygiene: practice and perspectives

**DOI:** 10.1186/1753-6561-5-S6-P109

**Published:** 2011-06-29

**Authors:** J Westbury

**Affiliations:** 1 1Faculty of Health Sciences, University of Southampton, Southampton, UK

## Introduction / objectives

Hand hygiene is considered the cornerstone of infection prevention practice, but previous studies demonstrate one group of healthcare professionals, doctors, have not achieved good levels of compliance in comparison to other staff groups. The aim of the research was to examine consultant doctors’ practice and perspectives of hand hygiene and explore their perceptions as leaders and role models, so as to identify strategies to improve compliance.

## Methods

The study design was based on naturalistic inquiry, focussing on the social constructions of participants. Consultant doctors (n=19) were observed during hospital ward rounds using both a national audit tool to assess hand hygiene compliance and recording of field notes. These same consultants, plus a further two (n=21), were interviewed individually to elicit their views. Data from interviews and field notes were analysed qualitatively using thematic content analysis.

## Results

Observations demonstrated high levels of hand hygiene compliance for high/medium risk activities, with low levels of compliance for low risk activities. Thematic content analysis revealed a strong belief by consultant doctors in the value of hand hygiene. However, a perceived conflict between political and scientific drivers of its promotion gave rise to confusion, frustration and a lack of engagement that created barriers to leadership and acting as a role model. Differing guidelines and audit tools that did not address levels of risk compounded the matter. However, they offered various recommendations to resolve the issues.

## Conclusion

Compliance with hand hygiene by consultant doctors is dependant on perceived level of risk. To promote leadership and role modelling it is critical to engage them, understand their views, employ their recommendations and recognise they are motivated by evidence-based rationales for practice rather than political mandates.

## Disclosure of interest

None declared.

